# The Standardisation of Sera

**Published:** 1921-03-12

**Authors:** 


### THE STANDARDISATION OF SERA.

It is not a little unfortunate that proposed legislative reforms involving highly technical matters should on occasion be fallaciously explained to the public. The latest misunderstanding has, however, found its way into the professional journals also, for whom surely there is the less excuse. At first sight, indeed, the proposal of State control for all biological medical substances implies so great an amount of departmental activity, so much expenditure, and at the same time further complication in the general practice of curative medicine that misgivings even among the initiated were inevitable. Direct inquiries, both at the Ministry of Health and from the manufacturers concerned, allow, however, but one interpretation of the process foreshadowed in the Departmental Committee's report.

To the practising doctor no hindrance can possibly arise. In any case he would, in this country, be absolutely assured of reliable "biologicals," as the *Lancet* names them, and in view of the increasing importation of insufficiently guaranteed substances, a measure of control will certainly prevent a dangerous situation from developing.

The suggestion of great and unnecessary expenditure can, for the benefit of the taxpaying public, also be contradicted with certainty. Admittedly, it is recommended that there should be a central laboratory wherein research and standardisation should be carried out. But the Medical Research Council are to be responsible, and they already possess not only the requisite organisation but the central laboratory as well. Also the testing done therein is to be confined to samples taken from makers' stocks or bought in the open market, exactly as with food inspection, and the primary responsibility of keeping up to standard is left to the manufacturers themselves. No one will object to licensing the manufacturers. Evidence was, in fact, taken from representatives of some of the largest and most reliable firms in the country, and all agreed that their premises, work carried on therein, and all records might well be subject to the necessary inspection and control. There is no suggestion that this measure is either intended or expected to check enterprise or hamper scientific progress.

Essentially the Committee recommend a scheme whereby the Government can deal with the matter but without creating a new institution. Let us remember, for example, the history of salvarsan. For some years the necessary biological work has been carried out by the scientific staff of the Medical Research Council. Now the Committee suggest that this Council should deal on similar biological lines with such other therapeutic substances as may be considered necessary. Whatever standards they prescribe, it can be left to the manufacturers to conform with them, and a minimum of inspection should, in the case of most of our established makers, be all that is necessary. No doubt there is some expense attached to any development of this kind, but obviously it would be but a fraction of that implied by the extremist critics on *The Times*.

The scheduling of these substances is not proposed to be entrusted to the Minister of Health or any of his Committees, but through the already existing committee of the Privy Council to the Medical Research Council itself. The public must, above everything, realise that this is not a bureaucratic interference, but simply a suggested mechanism whereby the best scientific control can be brought to bear upon the quality of certain medical substances which, for the complication of their manufacture and the urgent conditions in which they are often trusted, most certainly require it. There is no question of superseding famous pioneer laboratories. The staff of these institutions, both lay and scientific, are for obvious reasons in favour of a scheme which gives them, as well as the profession they serve, well-earned protection.

The Departmental Committee append to their report suggestions for a Draft Bill for the purpose of carrying their proposals into effect. The draft is, doubtless, the work of the Chairman, Sir Mackenzie Chalmers, whose skill as a Parliamentary draftsman is well known. So far as we are aware, however, the Ministry have not yet even signified their acceptance of the proposals it embodies, and for the Draft Bill itself Dr. Addison has certainly no responsibility whatever. But the Committee's scheme merits favourable consideration, and the improvement both in supply and quality of the biologicals, and the benefit to national health that their efficient use implies, easily outweigh the trifling expenditure involved.

